# Supplementary material for: A Cdc42-mediated supracellular network drives polarized forces and Drosophila egg chamber extension
Source: Nat Commun. 2020 Apr 21;11:1921. doi: 10.1038/s41467-020-15593-2 (PMC7174421; doi:10.1038/s41467-020-15593-2)
Supplement: Supplementary file 1 — Supplementary Information [file 41467_2020_15593_MOESM1_ESM.pdf]

a

b

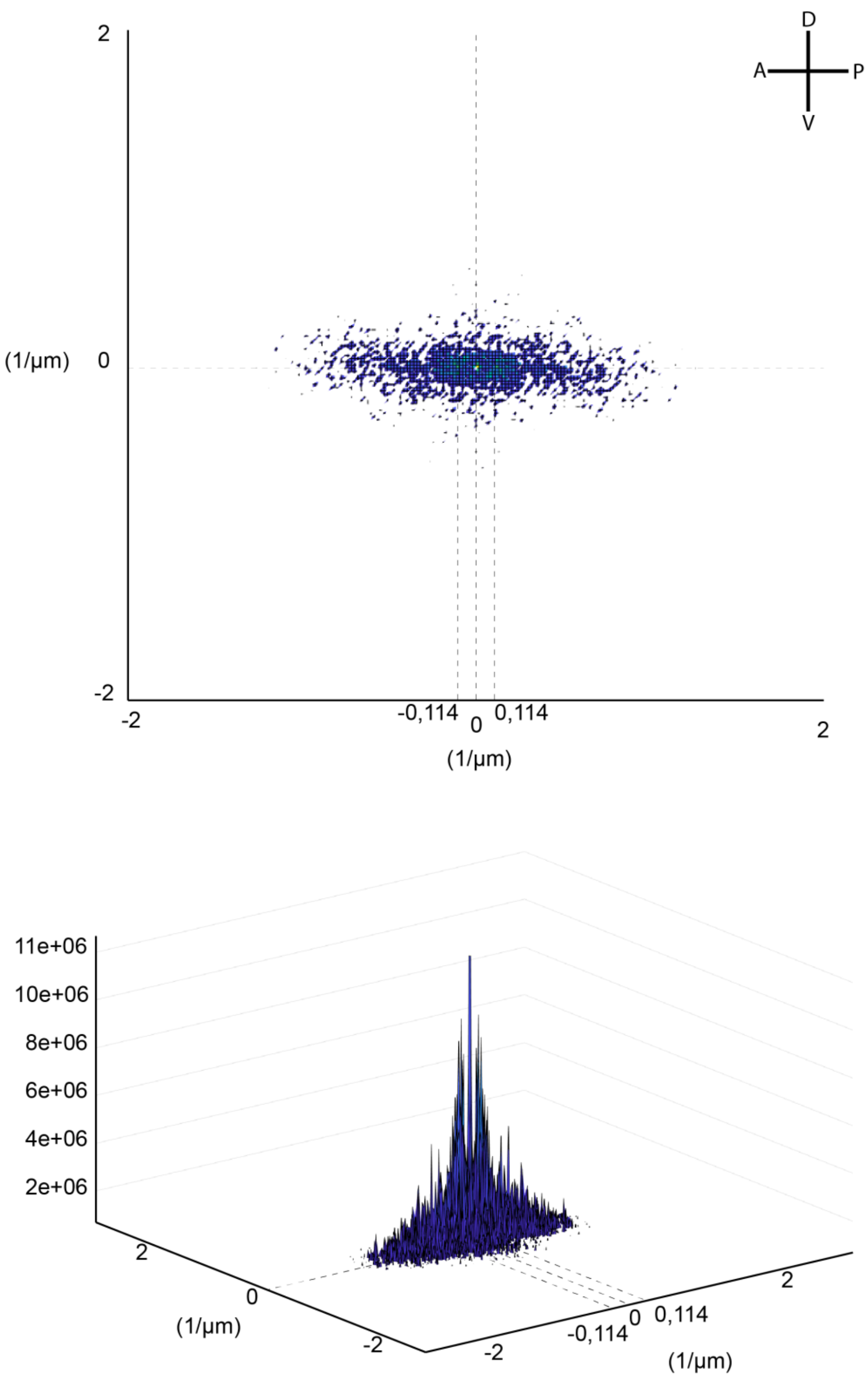

Supplementary Figure 1. F-actin organization revealed by Fourier transform.
Two-dimensional (**a**) and three-dimensional (**b**) plots of a 2D Fourier transform of the F-actin phalloidin staining image shown in **Fig. 1a**. Higher frequencies are revealed along the AP axis with picks at 0,114 (1/µm) frequency corresponding to ~9 µm cell width.

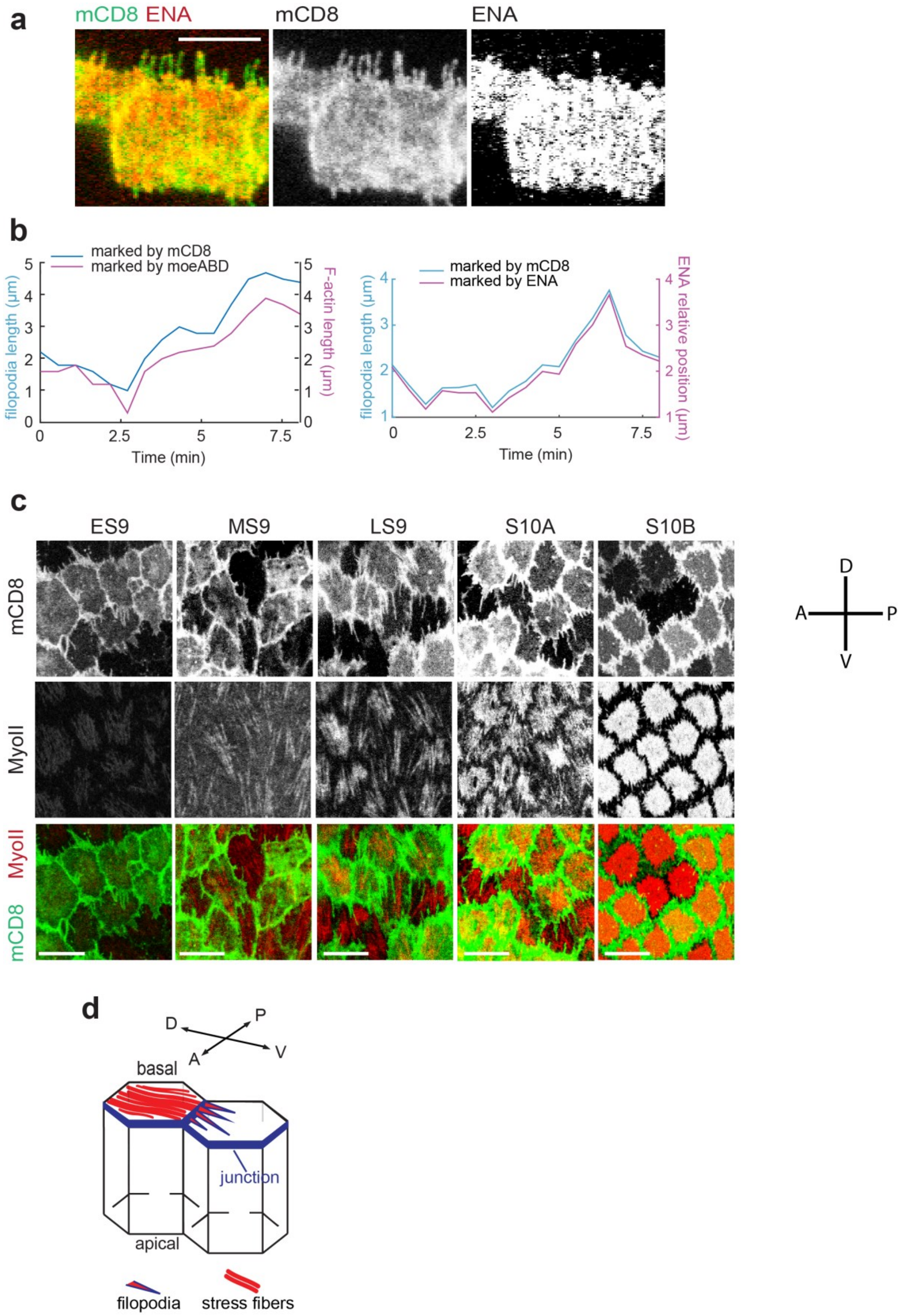

Supplementary Figure 2. Intercellular membrane protrusions are filopodia that extend starting from ES9 and retract during S10B.

a. Basal view of a follicle cell with expression of Enabled-RFP (red) and membrane label mCD8GFP (green) at S10A. **b.** Left panel: membrane (mCD8GFP) and F-actin (moesinABD)

length over time for a representative intercellular filopodium. Right panel: membrane length (mCD8GFP) and tip position (identified with the Enabled-RFP label) of a representative intercellular filopodium. **c.** Basal view of follicle cells at early stage 9 (ES9), middle stage 9 (MS9), late stage 9 (LS9), stage 10A (S10A) and stage 10B (S10B): MyoII-mCherry label (red) and stochastic expression of mCD8GFP (green). Arrows indicate the anterior-posterior and the ventral-dorsal directions. The results shown in (**a, c**) have been successfully repeated from the at least 4 independent experiments. **d.** Schematic diagram showing the basal distribution of intercellular filopodia and stress fibers in follicle cells. Scale bars are 10 μm.

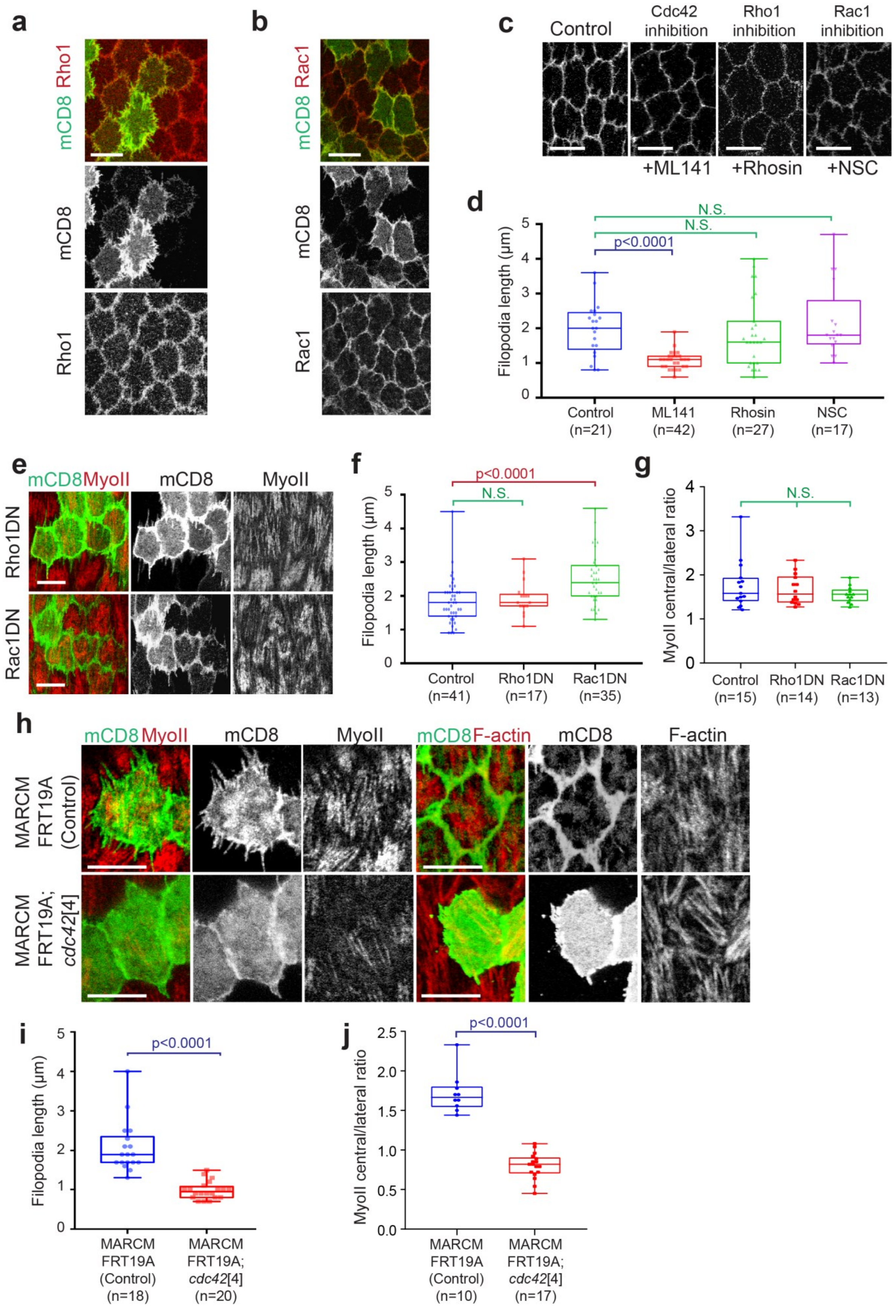

a
mCD8 Rho1
mCD8
Rho1
b
mCD8 Rac1
mCD8
Rac1
c
Control
Cdc42 inhibition
Rho1 inhibition
Rac1 inhibition
+ML141
+Rhosin
+NSC
d
Filopodia length (μm)
p<0.0001
N.S.
N.S.
Control (n=21)
ML141 (n=42)
Rhosin (n=27)
NSC (n=17)
e
mCD8MyoII
mCD8
MyoII
Rho1DN
Rac1DN
f
Filopodia length (μm)
p<0.0001
N.S.
Control (n=41)
Rho1DN (n=17)
Rac1DN (n=35)
g
MyoII central/lateral ratio
N.S.
Control (n=15)
Rho1DN (n=14)
Rac1DN (n=13)
h
mCD8MyoII
mCD8
MyoII
mCD8F-actin
mCD8
F-actin
MARCM FRT19A (Control)
MARCM FRT19A; cdc42[4]
i
Filopodia length (μm)
p<0.0001
MARCM FRT19A (Control) (n=18)
MARCM FRT19A; cdc42[4] (n=20)
j
MyoII central/lateral ratio
p<0.0001
MARCM FRT19A (Control) (n=10)
MARCM FRT19A; cdc42[4] (n=17)

Supplementary Figure 3. Cdc42 activity, rather than Rac1 or Rho1 activity, controls intercellular filopodia length and stress fiber distribution.

a, b. Confocal micrographs of Rho1-mCherry (**a**) and Rac1-GFP (**b**) in follicle cells with a stochastic expression of mCD8GFP (**a**) and mCD8RFP (**b)** clones, respectively. **c.** Basal view of follicle cells under different chemical inhibitor treatment conditions: ML141 (Cdc42 inhibitor), Rhosin (Rho1 inhibitor) and NSC23766 (Rac1 inhibitor). Membrane is labeled with Indy-GFP. The results shown in (**a-c**) have been successfully repeated from the at least 4 independent experiments. **d.** Average filopodia length per cell (from at least 3 egg chambers) for the n individual follicle cells under the indicated chemical treatment conditions. For the control vs. ML141 comparison: $p<0.0001$; for the control vs. Rhosin comparison: $p=0.3389$; for the control vs. NSC23766 comparison: $p=0.9595$; all by two-sided Mann-Whitney test. **e.** Basal view of follicle cells presenting Rho1DN (top panels) and Rac1DN (bottom panels) clones marked by mCD8GFP coexpression. MyoII (red) is visualized by using a MyoII-mCherry construct. **f.** Average filopodia length per cell (from at least 4 egg chambers) for the n individual follicle cells in Rho1DN, Rac1DN and wild type genetic backgrounds. For the control vs. Rho1DN comparison: $p=0.4172$; for the control vs. Rac1DN comparison: $p<0.0001$; both by two-sided Mann-Whitney test. **g.** Relative (central/lateral) distribution of MyoII intensity in the n individual follicle cells in Rho1DN, Rac1DN and wild type genetic backgrounds. For the control vs. Rho1DN comparison: $p=0.8382$; for the control vs. Rac1DN comparison: $p=0.4667$; both by two-sided Mann-Whitney test. **h.** Micrographs showing the basal side of follicle cell clones with the indicated genetic backgrounds, marked by mCD8GFP coexpression. MyoII and F-actin are visualized using MyoII-mCherry labelling and phalloidin staining, respectively. **i.** Average filopodia length per cell for the n individual follicle cells in control (FRT19A alone) and *cdc42*-LOF mutant genetic backgrounds. 2 egg chambers were analysed for the control and 5 for the mutant. $p<0.0001$ by two-sided Mann-Whitney test. **j.** Relative (central/lateral) distribution of MyoII in the n individual control (FRT19A alone) and *cdc42*-LOF mutant follicle cells. $p<0.0001$ by two-sided Mann-Whitney test. Scale bars are 10 µm in (**a, b, c, e, h**). In (**d, f, g, i, j**) boxes extend from the 25th to 75th percentiles, the mid line represents the median and the whiskers indicate the maximum and the minimum values.

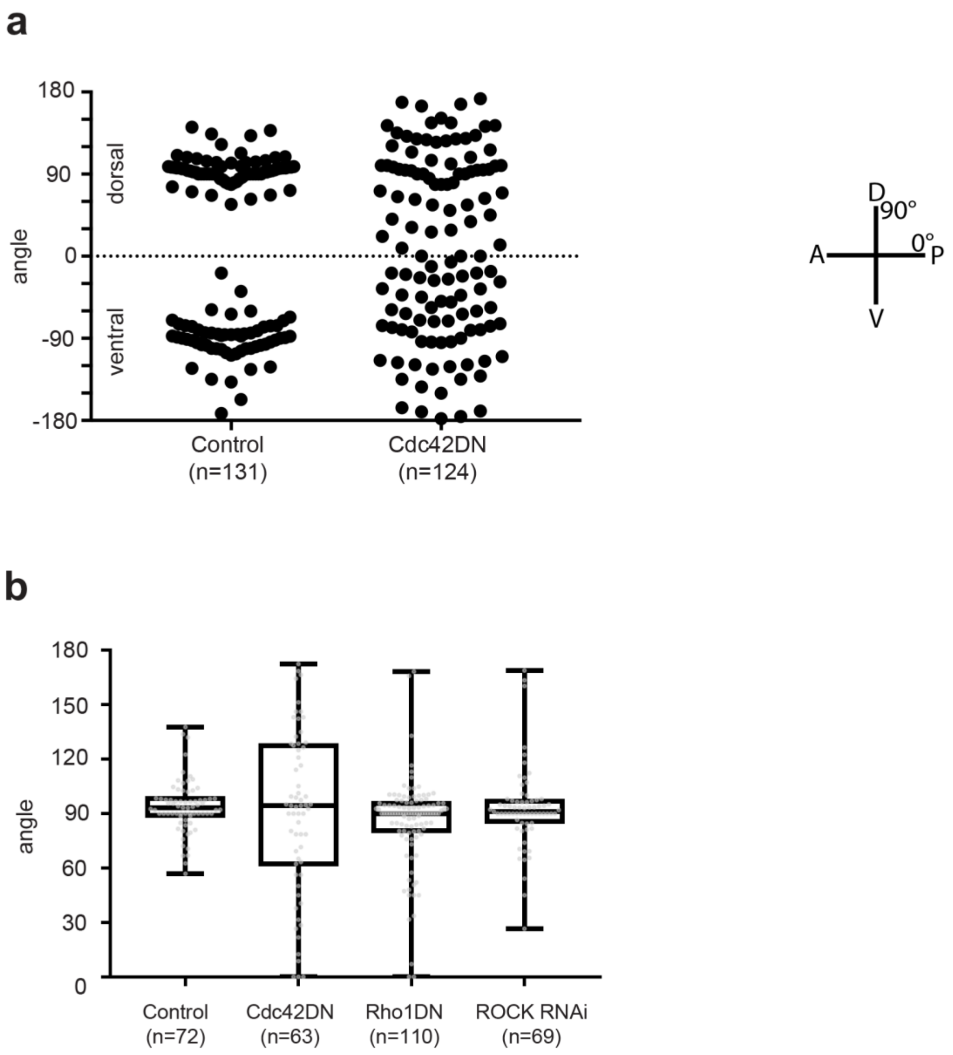

Supplementary Figure 4. Cdc42 activity, rather than Rho1 pathway activity, controls filopodia distribution mainly polarized along the DV axis.

a. Angle values measured between filopodium direction and the AP axis in control and Cdc42DN cells, for the n individual filopodia. **b.** Angle values measured between filopodium direction and the AP axis in control and cells expressing Cdc42DN, Rho1DN or ROCK RNAi transgenes, for the n individual filopodia (for this analysis, filopodia extending from cell dorsal junctions were used for simplicity). Standard deviation values are: 14.34 in control, 44.98 in Cdc42DN, 24.29 in Rho1DN and 22.03 in ROCK RNAi. In (**b**) boxes extend from the 25th to 75th percentiles, the mid line represents the median and the whiskers indicate the maximum and the minimum values.

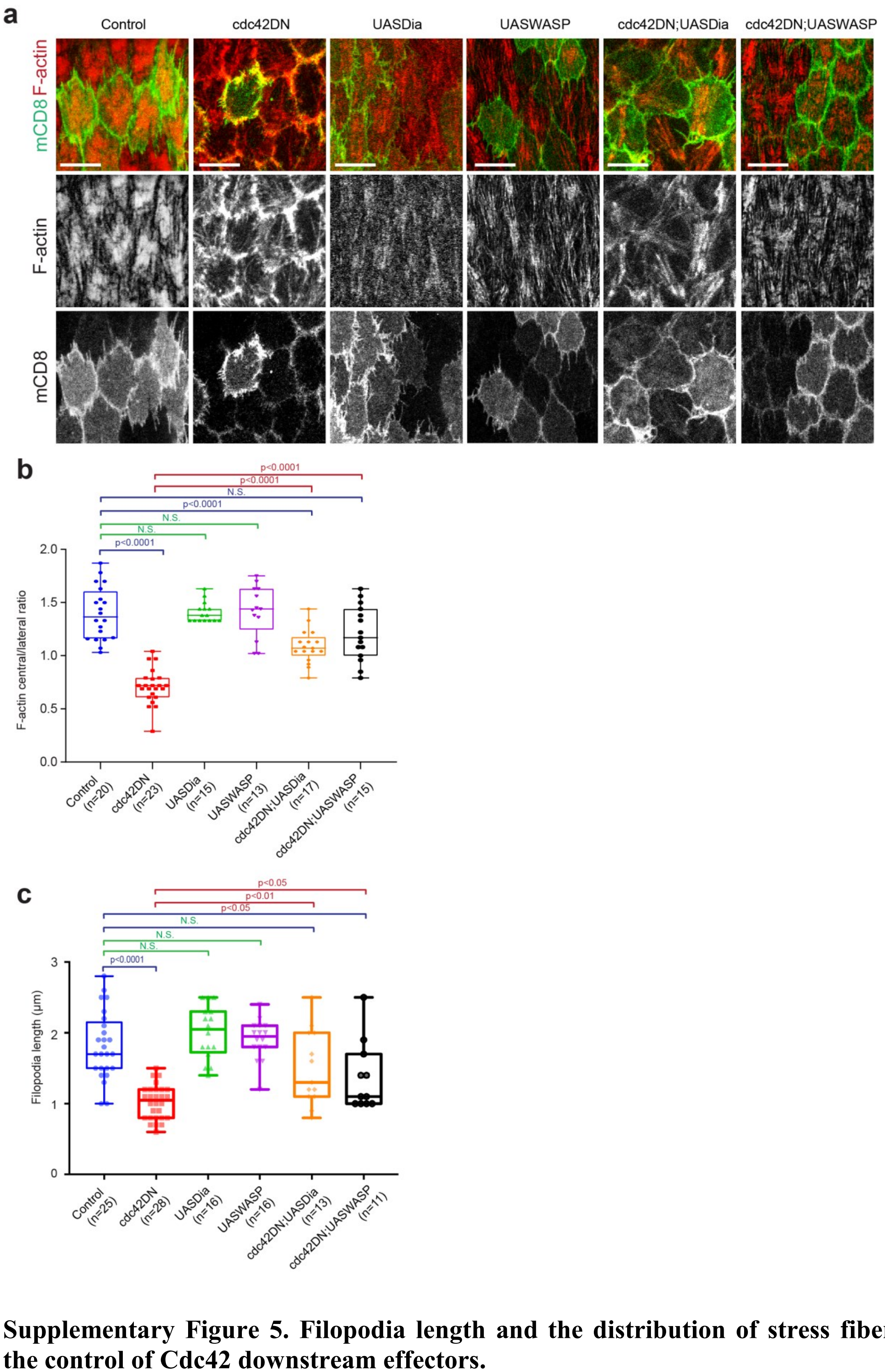

Supplementary Figure 5. Filopodia length and the distribution of stress fibers are under the control of Cdc42 downstream effectors.

a. Basal view of follicle cells showing clones expressing the indicated transgenes and coexpressing mCD8GFP. F-actin is labeled by phalloidin staining. Scale bar are 10 µm. **b.** Relative (central/lateral) distribution of F-actin intensity in the n individual follicle cells under the indicated genetic backgrounds. For the control vs. Cdc42DN comparison: $p<0.0001$; for the control vs. UAS-Dia comparison: $p=0.721$; for the control vs. UAS-WASP comparison: $p=0.7921$; for the control vs. Cdc42DN/UAS-Dia comparison: $p<0.0001$; for the control vs. Cdc42DN/UAS-WASP comparison: $p=0.0511$; for the Cdc42DN vs. Cdc42DN/UAS-Dia comparison: $p<0.0001$; for the Cdc42DN vs. Cdc42DN/UAS-WASP comparison: $p<0.0001$; all by two-sided Mann-Whitney test. **c.** Average filopodia length per cell (from at least 3 egg chambers) for the n individual follicle cells under the indicated genetic backgrounds. For the control vs. Cdc42DN comparison: $p<0.0001$; for the control vs. UAS-Dia comparison: $p=0.1707$; for the control vs. UAS-WASP comparison: $p=0.2899$; for the control vs. Cdc42DN/UAS-Dia comparison: $p=0.0827$; for the control vs. Cdc42DN/UAS-WASP comparison: $p=0.0104$; for the Cdc42DN vs. Cdc42DN/UAS-Dia comparison: $p=0.0029$; for the Cdc42DN vs. Cdc42DN/UAS-WASP comparison: $p=0.0402$; all by two-sided Mann-Whitney test. In (**b, c**) boxes extend from the 25th to 75th percentiles, the mid line represents the median and the whiskers indicate the maximum and the minimum values.

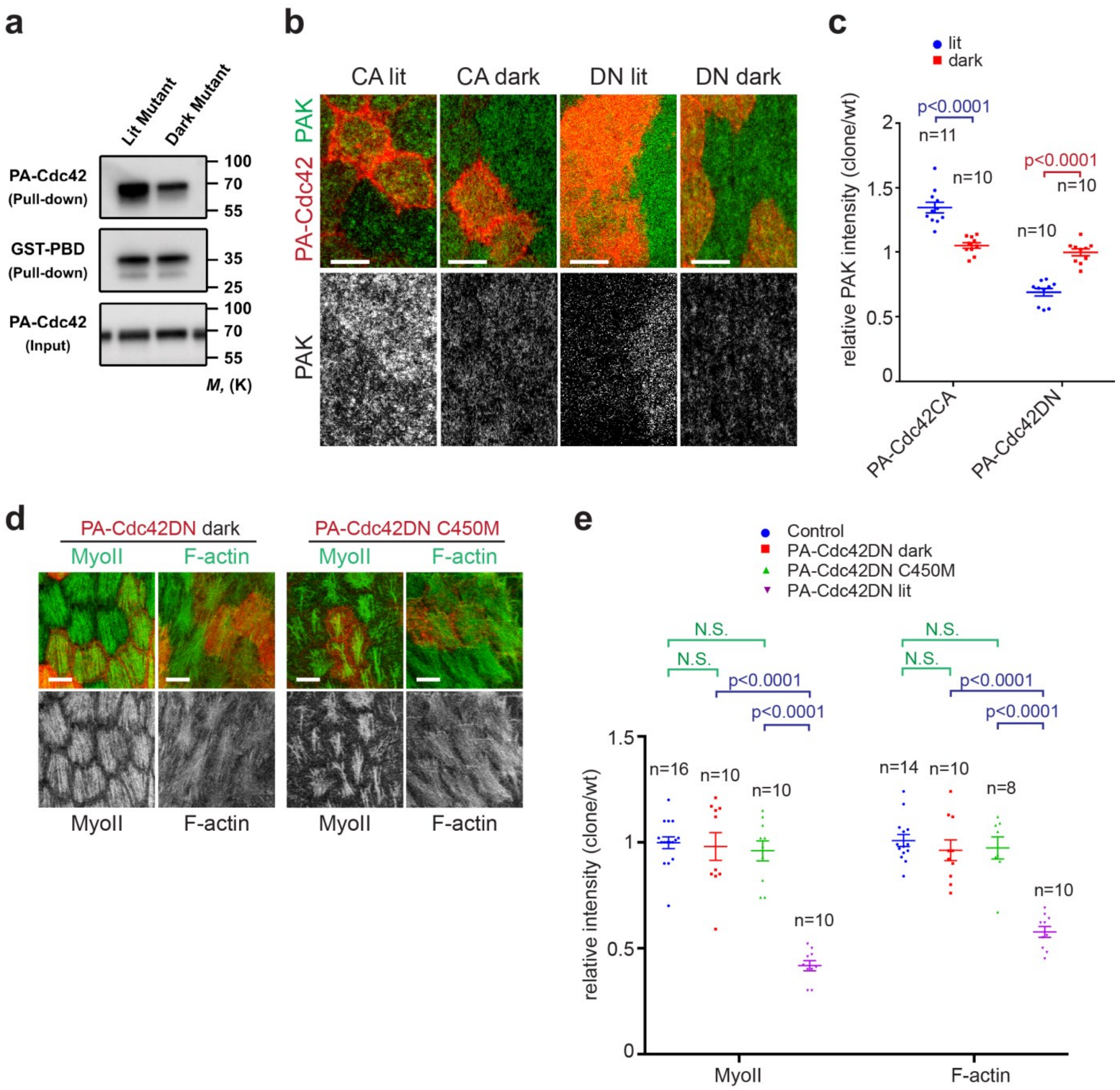

Supplementary Figure 6. Validation of the photoactivatable-Cdc42 (PA-Cdc42) optogenetic tool.

a. Light-dependent binding of PA-Cdc42 to PAK1-PBD. Lysates from HEK293 cells expressing either PA-Cdc42 lit state mutant (I531E/I539E) or dark state mutant (C450A, L514K, G528A, L531E, N538E) were incubated with GST-PAK1-PBD beads. GST pulldowns were analyzed by Western blotting as indicated. The PA-Cdc42 light-mimic mutant (I531E/I539E) strongly interacts with the GST-PBD, unlike the PA-Cdc42 dark-mimic mutant (C450A, L514K, G528A, L531E, N538E). Full scan figures for the western blots have been shown below in the supplementary figure 13. The results shown in (**a**) have been successfully repeated from the at least 3 independent experiments. **b.** Basal view of follicle cells showing clones expressing either PA-Cdc42CA or PA-Cdc42DN transgenes, under light or dark conditions, labeled with PAK-RBD-GFP. The PAK-RBD-GFP intensity signal is used to monitor and quantify the level of Cdc42 activity in these different conditions. **c.** Relative PAK-RBD-GFP intensity (Clone/WT) at

the basal side of the n individual follicle cells under the indicated conditions. For the PA-Cdc42CA lit vs. dark comparison: $p<0.0001$; for the PA-Cdc42DN lit vs. dark comparison: $p<0.0001$; both by two-sided Mann-Whitney test. **d.** Basal view of follicle cells showing clones expressing either PA-Cdc42DN or PA-Cdc42DN C450M transgenes, under dark conditions. MyoII and F-actin have been labeled using MyoII-GFP and UtrABD-GFP respectively. **e.** Relative (clone/wild type) intensity of basal MyoII and F-actin in the n individual follicle cells under the indicated genetic backgrounds. For MyoII signal, the control vs. PA-Cdc42DN dark comparison: $p>0.9999$, the control vs. PA-Cdc42DN C450M comparison: $p=0.8047$, for the PA-Cdc42DN dark vs. PA-Cdc42DN lit comparison: $p<0.0001$, for the PA-Cdc42DN C450M vs. PA-Cdc42DN lit comparison: $p<0.0001$; for F-actin signal, the control vs. PA-Cdc42DN dark comparison: $p=0.2522$, the control vs. PA-Cdc42DN C450M comparison: $p=0.8284$, for the PA-Cdc42DN dark vs. PA-Cdc42DN lit comparison: $p<0.0001$, for the PA-Cdc42DN C450M vs. PA-Cdc42DN lit comparison: $p<0.0001$; all by two-sided Mann-Whitney test. Data are presented as mean values +/- SEM. Scale bars are 5 µm in (**b, d**).

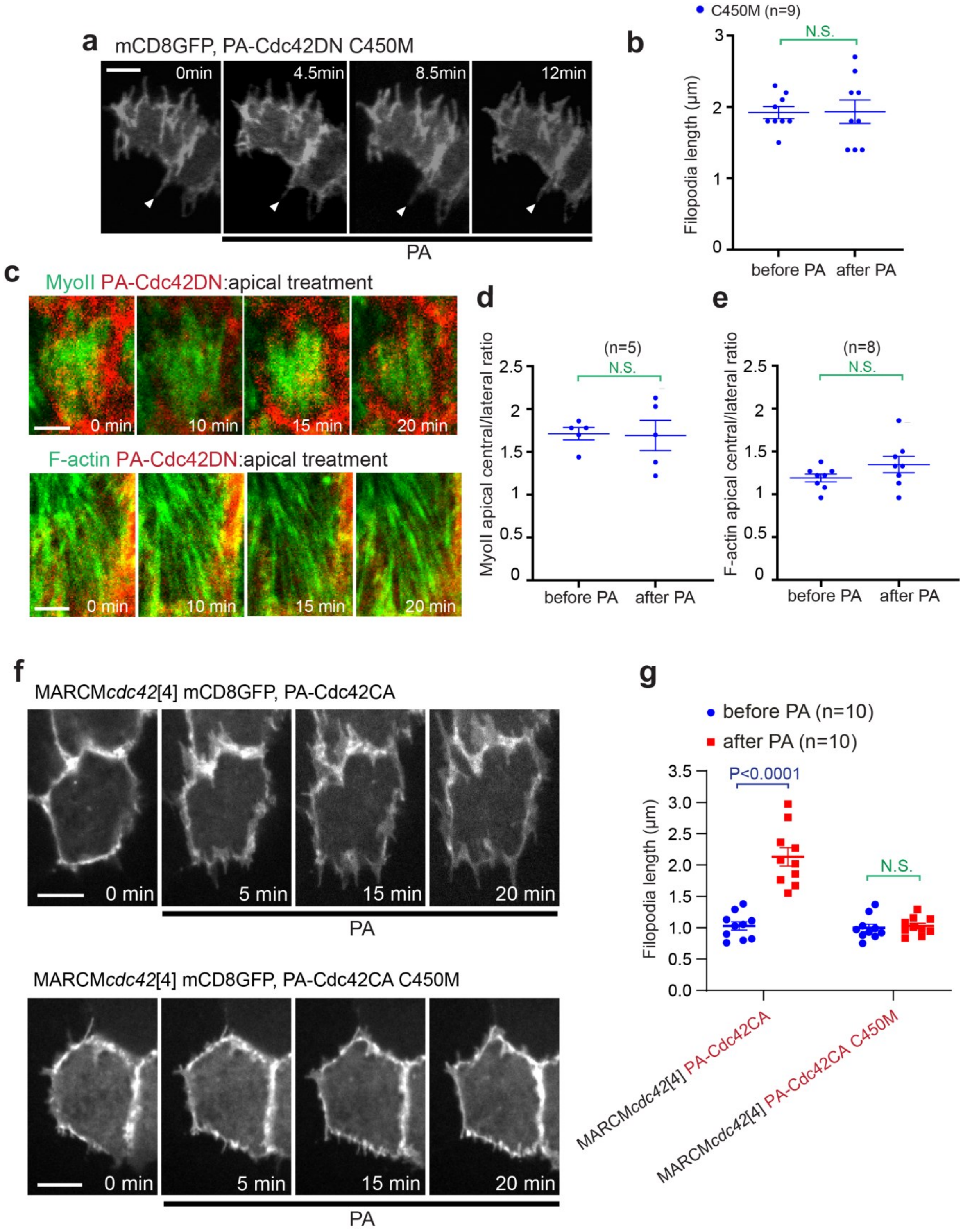

Supplementary Figure 7. Control experiments for the photoactivatable-Cdc42 (PA-Cdc42) optogenetic tool.

a. Time-lapse series of a representative mCherry-tagged PA-Cdc42DN C450M- expressing follicle cell, coexpressing mCD8GFP. PA indicates the time of photoactivation. Arrow heads indicate filopodia. **b.** Average filopodia length per cell (from 5 egg chambers) for the n

individual follicle cells under the indicated genetic background before and 10-15 minutes after photoactivation. p=0.9065 by two-sided Mann-Whitney test. **c.** Time-lapse series of a representative mCherry-tagged PA-Cdc42DN-expressing follicle cell labelled with MyoII-GFP (upper panels) and UtrABD-GFP (lower panels), respectively. Micrographs have been captured at the basal domain, while photoactivation was induced at the apical domain of follicle cells. **d, e.** Relative (central/lateral) distribution of MyoII (**d**) and F-actin (**e**) before and 20-25 minutes after photoactivation at the apical domain of the n individual PA-Cdc42DN-expressing follicle cells. For the MyoII signal relative distribution comparison: p=0.9127; for the F-actin signal relative distribution comparison: p=0.182; both by two-sided Mann-Whitney test. **f.** Time-lapse series of representative PA-Cdc42CA- or PA-Cdc42CA C450M- expressing follicle cell with *cdc42*[4] mutant genetic background, labeled with mCD8GFP. PA indicates the time of photoactivation. **g.** Average filopodia length per cell in the n individual PA-Cdc42CA or PA-Cdc42CA C450M clone cells before and 15-20 minutes after photoactivation (from at least 6 egg chambers for both conditions). For the *cdc42*[4]/PA-Cdc42CA before- vs. after- photoactivation comparison: p<0.0001; for the *cdc42*[4]/PA-Cdc42CA C450M before- vs. after- photoactivation comparison: p=0.5423; both by two-sided Mann-Whitney test. Data are presented as mean values +/- SEM. Scale bars are 5 μm in (**a, c, f**).

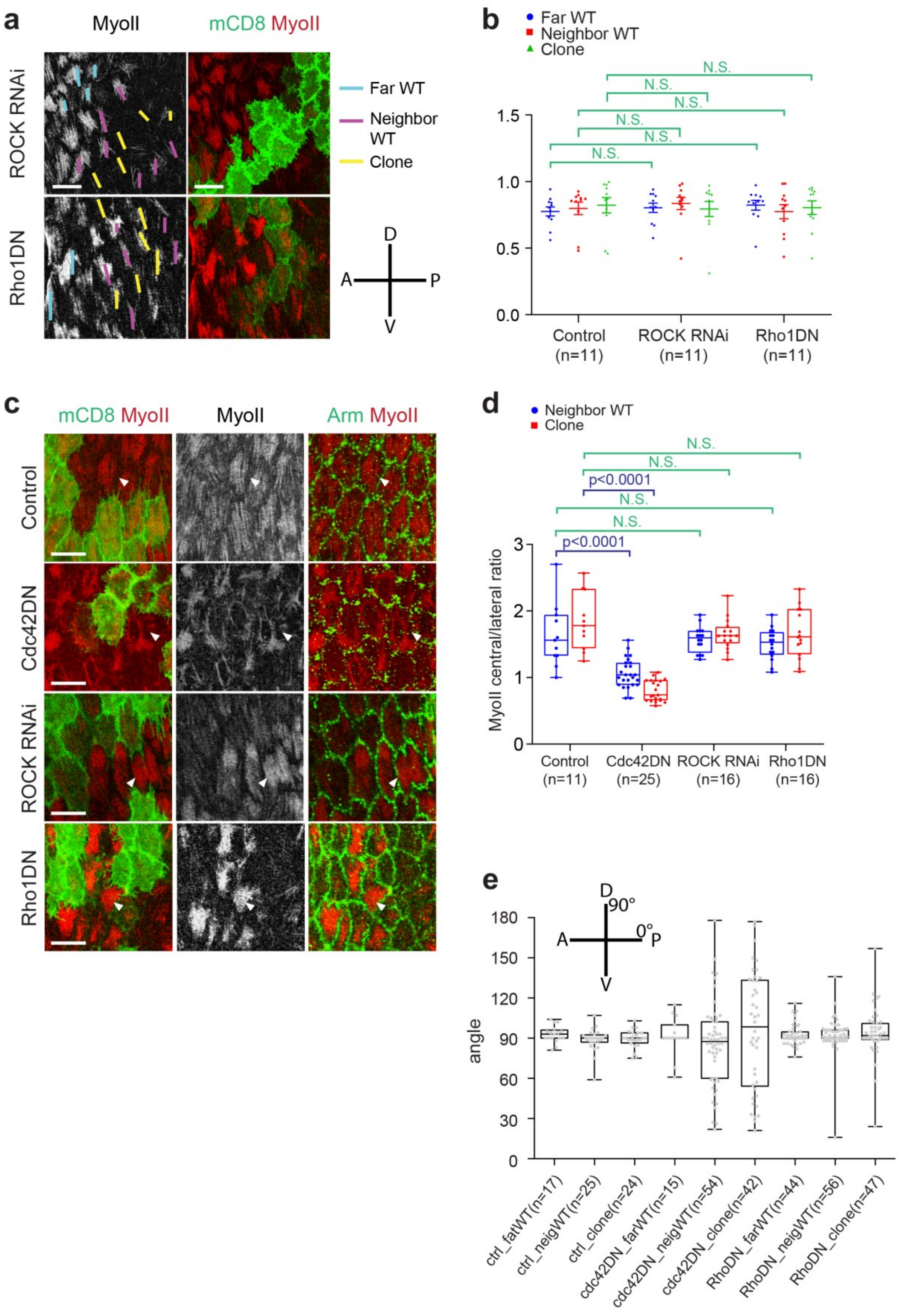

Supplementary Figure 8. Cdc42, but not Rho1 or ROCK, controls the stress fiber distribution and polarity in a non-cell autonomous manner.

a. Basal view of follicle cell clones expressing ROCK RNAi and Rho1DN transgene and coexpressing mCD8GFP. MyoII is labeled using a MyoII-mCherry construct. Yellow lines,

purple lines and blue lines mark the orientation of the basal stress fibers in the clone cells, in the wild type cells neighboring the clone cells and in the wild type cells not neighboring the clone cells, respectively. Arrows indicate the anterior-posterior and ventral-dorsal axes. **b.** Order parameter of clones, wild type cells neighboring the clones, and wild type cells not neighboring the clones respectively, measured from the n egg chambers with the indicated genetic backgrounds shown in (**a**). Data are presented as mean values +/- SEM. For the far WT cells, the control vs. ROCK RNAi comparison: p=0.1932, the control vs. Rho1DN comparison: p=0.1713; for the neighbor WT cells, the control vs. ROCK RNAi comparison: p=0.3653, the control vs. Rho1DN comparison: p=0.6063; for the clone cells, the control vs. ROCK RNAi comparison: p=0.6522, the control vs. Rho1DN comparison: p=0.6994; all by two-sided Mann-Whitney test. **c.** Basal view of follicle cell clones expressing Cdc42DN, ROCK RNAi and Rho1DN transgenes and coexpressing mCD8GFP. MyoII is labeled using a MyoII-mCherry construct. The basal junctional cortex is labeled by Armadillo staining. Arrow heads indicate one wild type cell neighboring the indicated clone cells. **d.** Relative (central/lateral) distribution of MyoII intensity in the n individual clones, and in the n individual wild type cells neighboring the clones, respectively. For the neighbor WT cells, the control vs. Cdc42DN comparison: p<0.0001, the control vs. ROCK RNAi comparison: p=0.6601, the control vs. Rho1DN comparison: p=0.6705; for the clone cells, the control vs. Cdc42DN comparison: p<0.0001, the control vs. ROCK RNAi comparison: p=0.2646, the control vs. Rho1DN comparison: p=0.2867; all by two-sided Mann-Whitney test. Scale bars are 10 µm in (**a, c**). **e.** Angle values measured between stress fiber direction and the AP axis in control and cells expressing Cdc42DN or Rho1DN, for the n individual cells. In (**d, e**) boxes extend from the 25th to 75th percentiles, the mid line represents the median and the whiskers indicate the maximum and the minimum values.

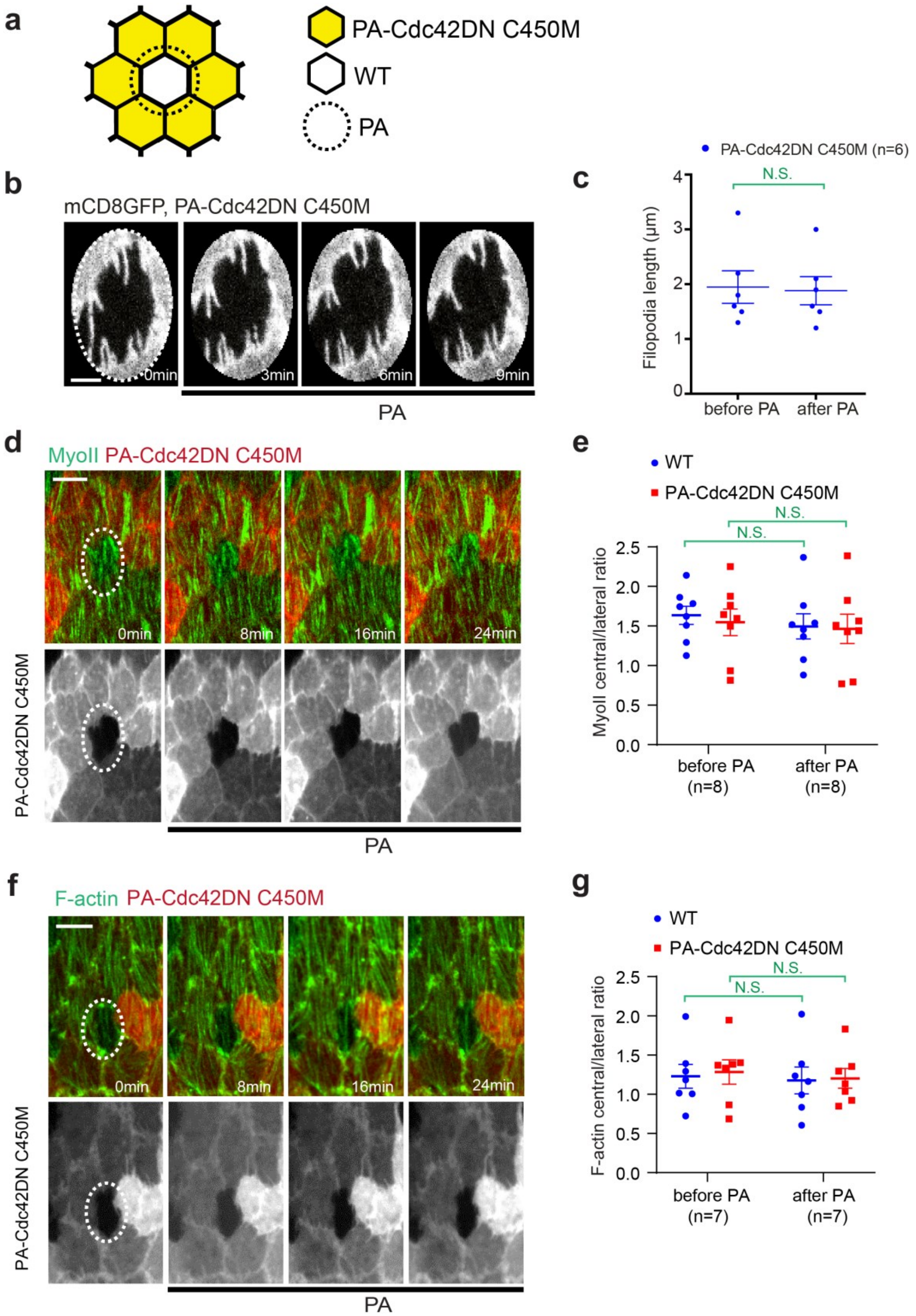

Supplementary Figure 9. Cdc42 photoactivation control experiments for the non-cell autonomous effect.

a. Schematic diagram showing the region of photoactivation (PA, dashed circle) in experiments where one wild type cell is surrounded by PA-Cdc42DN C450M-expressing clones. Only the junctional cortexes of PA-Cdc42DN C450M clones facing the wild type cells are photoactivated.

b, d, f. Time-lapse series showing the experiment represented in (**a**) for follicle cells labeled with mCD8GFP (**b**), MyoII-GFP (**d**) and UtrABD-GFP (**f**). Dashed ellipses indicate the photoactivated region. PA indicates the time of photoactivation. **c.** Average filopodia length per cell (from 4 egg chambers) for the n individual samples as shown in (**a**), before and 10-15 minutes after photoactivation. p=0.9156 by two-sided Mann-Whitney test. **e, g.** Relative (central/lateral) distribution of MyoII (**e**) and F-actin (**g**) in the n individual wild type and PA-Cdc42DN C450M-expressing cells before and 25-30 minutes after photoactivation. For the relative distribution of MyoII signal, the WT before- vs. after- photoactivation: p=0.3823, the PA-Cdc42DN C450M before- vs. after- photoactivation: p=0.4418; for the relative distribution of F-actin signal, the WT before- vs. after- photoactivation: p=0.7104, the PA-Cdc42DN C450M before- vs. after- photoactivation: p=0.4557; all by two-sided Mann-Whitney test. Data are presented as mean values +/- SEM. Scale bars are 5 μm in (**b**) and 10 μm in (**d, f**).

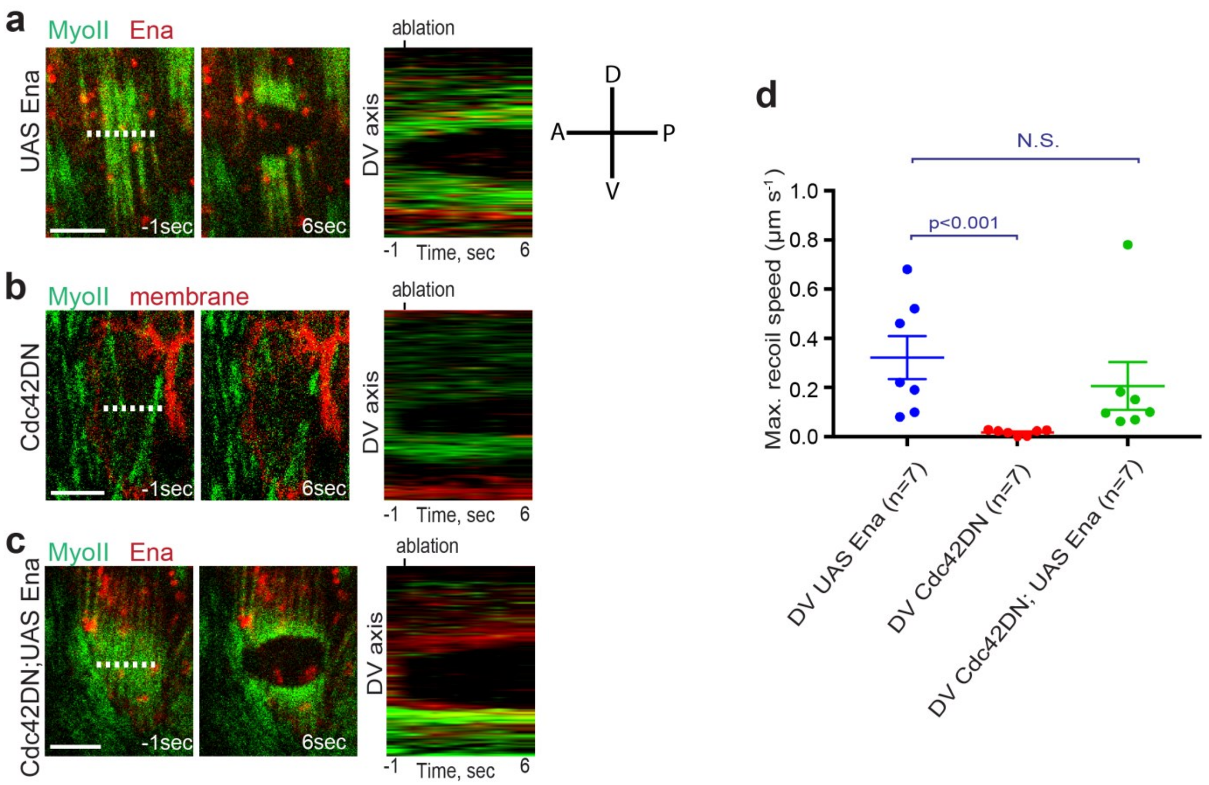

Supplementary Figure 10. Cortical tension anisotropy is under the control of Cdc42 downstream effector Ena.

a-c. Time-lapse series of a representative cell expressing UAS EnaRFP (**a**), Cdc42DN marked by mCD8GFP coexpression (**b**) or coexpressing UAS EnaRFP and Cdc42DN (**c**), before (panel on the left) and after ablation (indicated by the dashed line) of the basal actomyosin network along the AP axis. MyoII is visualized by using a MyoII-GFP (**a**, **c**) or MyoII-mCherry (**b**) construct. Right panel: kymographs illustrating network recoil along the DV axis after ablation. **d.** Maximum recoil speed after ablations in the n individual clones under the indicated genetic background. Data are presented as mean values +/- SEM. For the UAS-Ena vs. Cdc42DN DV comparison: $p=0.0006$; for the UAS-Ena vs. UAS-Ena/Cdc42DN DV comparison: $p=0.2086$; both by two-sided Mann-Whitney test. Scale bars are 5 μm in (**a-c**).

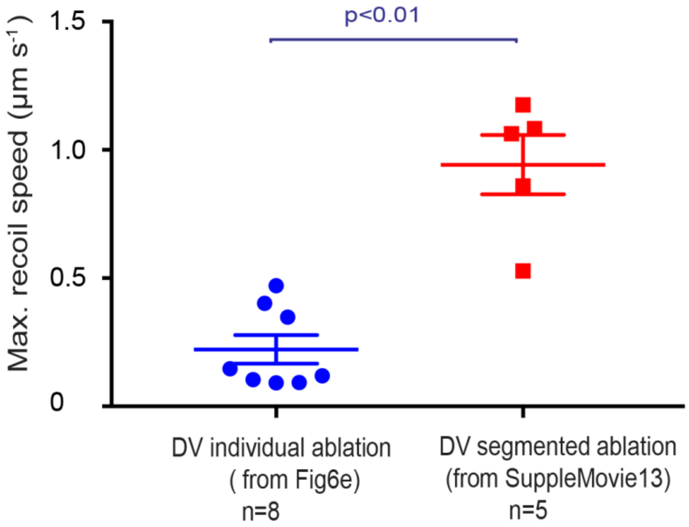

Supplementary Figure 11. Comparison between single and multiple parallel stress fiber network ablations.

Maximum recoil speed of the basal actomyosin network quantified for the n individual single cells after ablations along the AP axis in the case of individual cell ablations (data from Fig. 6e) and in the case of segmented tissue scale ablation (Supplementary Movie 13). Data are presented as mean values +/- SEM. p=0.0016 by two-sided Mann-Whitney test.

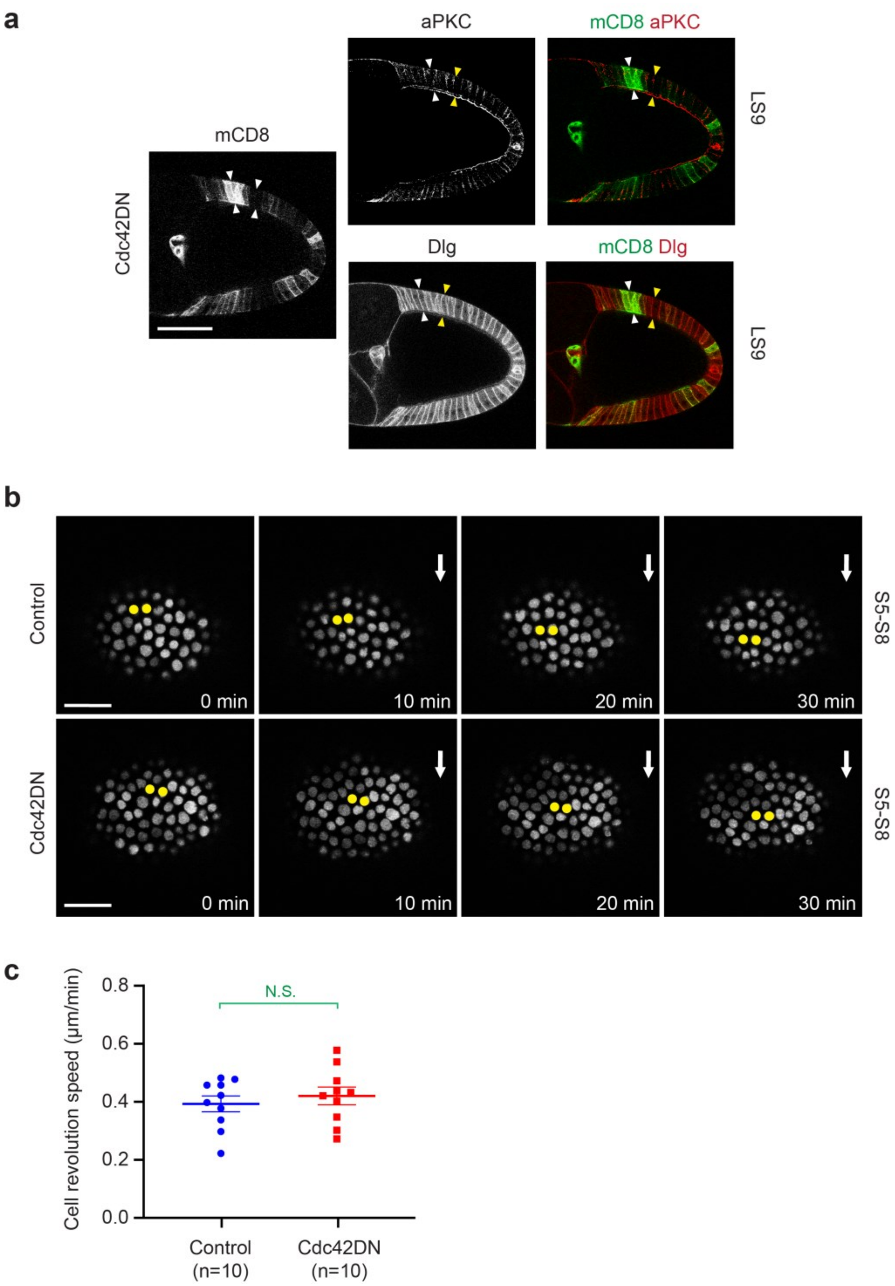

Supplementary Figure 12. Cdc42 inhibition does not affect follicle cell apical-basal polarity nor egg chamber revolution.

a. Sagittal plane of a LS9 egg chamber showing clones expressing the Cdc42DN transgene and coexpressing mCD8GFP. Arrow heads indicate clone (white) and wild type (yellow) follicle cells. Apical and basolateral polarities are labeled using aPKC and Dlg antibody staining, respectively. Scale bar is 50 µm. The results have been successfully repeated from the at least 3 independent experiments. **b.** Time-lapse series of a wild type (control) and a Cdc42DN representative egg chambers labelled with nuclear dsRed. Two cells in each egg chamber have been marked in yellow to emphasize tissue rotation. Arrows indicate rotation direction. Scale bar is 20 µm. **c.** Follicle cell revolution speed in the n individual egg chambers under the wild type

and Cdc42DN genetic backgrounds. Data are presented as mean values +/- SEM. p=0.6696 by two-sided Mann-Whitney test.

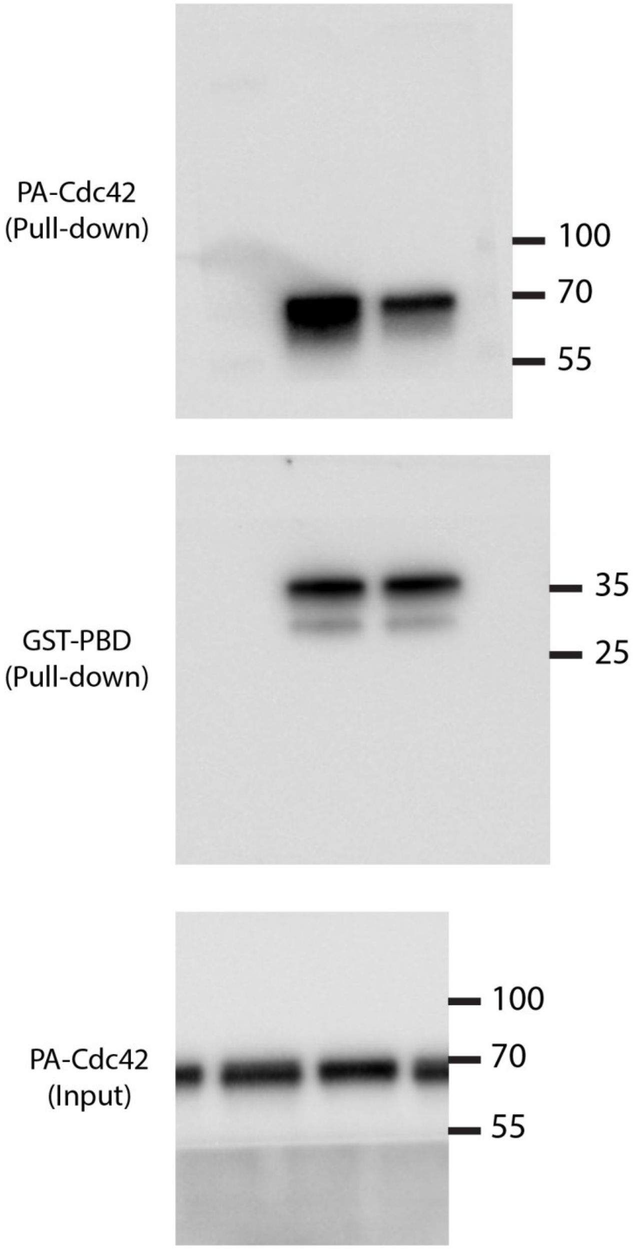

Supplementary Figure 13. Original data of western blot analyses shown in supplementary figure 6a. The results have been successfully repeated from the at least 3 independent experiments.

Fly stock	Origin	Reference
Sqh::RLCmyosinII–GFP	Eric E. Wieschaus	1
Sqh::RLCmyosinII–mCherry	Eric E. Wieschaus	1
Sqh::UtrABD–GFP	Thomas Lecuit	2
UAS-moesinABD-mCherry	Brooke M. McCartney	3
Endo Ecad GFP	Yohanns Bellaïche	
UAS-WASP-GFP	Arno Müller	
UAS-*Dia*^{RNAi}	#103914, VDRC	
UAS-*ROCK*^{RNAi}	#3793, VDRC	
Sqh::Cdc42-mCherry	#42236, BDSC	4
Rac1-GFP	#52283, BDSC	4
Rho1-mCherry	#52281, BDSC	4
Indy-GFP	#50860, BDSC	
UAS-Dia-GFP	#56751, BDSC	
UAS-Ena-RFP	#58744, BDSC	
UAS-*Ena*^{RNAi}	#31582, BDSC	
UAS-*WASP*^{RNAi}	#51802, BDSC	
Sqh::PAK-RBD-GFP	#56549, BDSC	
cdc42[4] P{neoFRT}19A/FM6	#9106, BDSC	5
UAS-mCD8GFP	#5130, BDSC	
UAS-mCD8RFP	#27399, BDSC	
UAS-Cdc42DN	#6288, BDSC	
UAS-Rho1DN	#7327, BDSC	
UAS-Rac1DN	#6292, BDSC	
UAS-dsRed	#6282, BDSC	
CoinFLP-LexA::GAD.GAL4	#58755, BDSC	
P[*hsp*70-flp]	#23647, BDSC	
*Ay*Gal4	#3953, BDSC	
Act5C-Gal4	#4414, BDSC	
P{neoFRT}19A	#1709, BDSC	
P{hsFLP}1, P{tubP-GAL80}LL1 P{neoFRT}19A	#5132, BDSC	
tubGal4	#5138, BDSC	
UAS-PA-Cdc42DN	This study	
UAS-PA-Cdc42DN C450M	This study	
UAS-PA-Cdc42CA	This study	
UAS-PA-Cdc42CA C450M	This study	

Supplementary Table 1. The list of fly strains. VDRC is Vienna *Drosophila* Resource center, BDSC is Bloomington *Drosophila* stock center.

Drug/antibody	Origin	Reference
Cdc42 inhibitor ML141	Sigma	6
Rac inhibitor NSC23766	Sigma	7
Rho1 inhibitor Rhosin	Merk	8
anti-WASP antibody	P5E1, DSHB	
anti-Ena antibody	5G2, DSHB	
anti-Dlg	4F3, DSHB	
anti-Arm	N27A1, DSHB	
anti-aPKC	sc-216, Santa Cruz biotechnology	
Anti-Dia antibody	gift from Steve Wasserman	
Alexa-561-conjugated phalloidin	Invitrogen	

DSHB is Developmental Studies Hybridoma Bank

Supplementary Table 2. The list of drugs and antibodies.

Supplementary References

1. Martin, A.C., Kaschube, M. & Wieschaus, E.F. Pulsed contractions of an actin-myosin network drive apical constriction. *Nature* **457**, 495-499 (2009).
2. Rauzi, M., Lenne, P.F. & Lecuit, T. Planar polarized actomyosin contractile flows control epithelial junction remodelling. *Nature* **468**, 1110-1114 (2010).
3. Vilmos, P. *et al.* Live imaging reveals that the Drosophila actin-binding ERM protein, moesin, co-localizes with the mitotic spindle. *Eur J Cell Biol* **88**, 609-619 (2009).
4. Abreu-Blanco, M.T., Verboon, J.M. & Parkhurst, S.M. Coordination of Rho family GTPase activities to orchestrate cytoskeleton responses during cell wound repair. *Curr Biol* **24**, 144-155 (2014).
5. Fehon, R.G., Oren, T., LaJeunesse, D.R., Melby, T.E. & McCartney, B.M. Isolation of mutations in the Drosophila homologues of the human Neurofibromatosis 2 and yeast CDC42 genes using a simple and efficient reverse-genetic method. *Genetics* **146**, 245-252 (1997).
6. Hong, L. *et al.* Characterization of a Cdc42 protein inhibitor and its use as a molecular probe. *J Biol Chem* **288**, 8531-8543 (2013).
7. Gao, Y., Dickerson, J.B., Guo, F., Zheng, J. & Zheng, Y. Rational design and characterization of a Rac GTPase-specific small molecule inhibitor. *Proc Natl Acad Sci U S A* **101**, 7618-7623 (2004).
8. Shang, X. *et al.* Small-molecule inhibitors targeting G-protein-coupled Rho guanine nucleotide exchange factors. *Proc Natl Acad Sci U S A* **110**, 3155-3160 (2013).
