# Supplementary material for: A Cdc42-mediated supracellular network drives polarized forces and Drosophila egg chamber extension
Source: Nat Commun. 2020 Apr 21;11:1921. doi: 10.1038/s41467-020-15593-2 (PMC7174421; doi:10.1038/s41467-020-15593-2)
Supplement: Supplementary file 3 — Description of Additional Supplementary Information [file 41467_2020_15593_MOESM3_ESM.pdf]

Description of Additional Supplementary Files

File Name: Supplementary Movie 1
Description: Time-lapse of a representative wild type follicle cell labeled with MyoII-mCherry. Scale bar 5 µm.

File Name: Supplementary Movie 2
Description: Time-lapse of a representative Cdc42DN-expressing follicle cell labeled with mCD8 GFP and MyoII-mCherry. Scale bar 5 µm.

File Name: Supplementary Movie 3
Description: Time-lapse of a representative Rho1DN-expressing follicle cell labeled with mCD8 GFP and MyoII-mCherry. Scale bar 5 µm.

File Name: Supplementary Movie 4
Description: Time-lapse of a representative PA-Cdc42DN-expressing follicle cell labeled with MyoII-GFP and photoactivated (the photoactivation region covers the entire field of view). Scale bar 5 µm.

File Name: Supplementary Movie 5
Description: Time-lapse of a representative PA-Cdc42DN C450M-expressing follicle cell labeled with MyoII-GFP and photoactivated (the photoactivation region covers the entire field of view). Scale bar 5 µm.

File Name: Supplementary Movie 6
Description:. Time-lapse of a representative PA-Cdc42DN-expressing follicle cell labeled with UtrABD-GFP and photoactivated (the photoactivated region covers the full field of view). Scale bar 5 µm.

File Name: Supplementary Movie 7
Description: Time-lapse of a representative PA-Cdc42DN C450M-expressing follicle cell labeled with UtrABD-GFP and photoactivated (the photoactivated region covers the full field of view). Scale bar 5 µm.

File Name: Supplementary Movie 8
Description: Time-lapse of a representative mCD8GFP-expressing follicle cell labeled with MyoII-mCherry. Laser ablation was performed along the AP axis. Scale bar 5 µm.

File Name: Supplementary Movie 9
Description: Time-lapse of a representative mCD8GFP-expressing follicle cell labeled with MyoII-mCherry. Laser ablation was performed along the DV axis. Scale bar 5 µm.

File Name: Supplementary Movie 10
Description: Time-lapse of a representative Cdc42DN-expressing follicle cell labeled with mCD8 GFP and MyoII-mCherry. Laser ablation was performed along the AP axis. Scale bar 5 µm.

File Name: Supplementary Movie 11
Description: Time-lapse of a representative mCD8GFP-expressing egg chamber labelled with MyoII-mCherry. Laser ablation was performed along the AP axis. Scale bar 10 µm.

File Name: Supplementary Movie 12
Description: Time-lapse of a representative mCD8GFP-expressing follicle cell labelled with MyoII-mCherry. Laser ablation was performed along the DV axis. Scale bar 10 µm.

File Name: Supplementary Movie 13
Description: Time-lapse of a representative mCD8GFP-expressing egg chamber labelled with MyoII-mCherry. Laser ablation was performed over a segmented line along the AP axis. Scale bar 10 µm.

File Name: Supplementary Movie 14
Description: Time-lapse of a representative mCD8GFP-expressing egg chamber labelled with MyoII-mCherry. Laser ablation was performed along the AP axis. Arrow heads indicate the current position of the filopodium. Scale bar 10 µm.
